# In Vivo Generation of Neurotoxic Prion Protein: Role for Hsp70 in Accumulation of Misfolded Isoforms

**DOI:** 10.1371/journal.pgen.1000507

**Published:** 2009-06-05

**Authors:** Pedro Fernandez-Funez, Sergio Casas-Tinto, Yan Zhang, Melisa Gómez-Velazquez, Marco A. Morales-Garza, Ana C. Cepeda-Nieto, Joaquín Castilla, Claudio Soto, Diego E. Rincon-Limas

**Affiliations:** 1 Department of Neurology, University of Texas Medical Branch, Galveston, Texas, United States of America; 2 Department of Neuroscience and Cell Biology, University of Texas Medical Branch, Galveston, Texas, United States of America; 3 Mitchell Center for Neurodegenerative Disorders, University of Texas Medical Branch, Galveston, Texas, United States of America; University of Minnesota, United States of America

## Abstract

Prion diseases are incurable neurodegenerative disorders in which the normal cellular prion protein (PrP^C^) converts into a misfolded isoform (PrP^Sc^) with unique biochemical and structural properties that correlate with disease. In humans, prion disorders, such as Creutzfeldt-Jakob disease, present typically with a sporadic origin, where unknown mechanisms lead to the spontaneous misfolding and deposition of wild type PrP. To shed light on how wild-type PrP undergoes conformational changes and which are the cellular components involved in this process, we analyzed the dynamics of wild-type PrP from hamster in transgenic flies. In young flies, PrP demonstrates properties of the benign PrP^C^; in older flies, PrP misfolds, acquires biochemical and structural properties of PrP^Sc^, and induces spongiform degeneration of brain neurons. Aged flies accumulate insoluble PrP that resists high concentrations of denaturing agents and contains PrP^Sc^-specific conformational epitopes. In contrast to PrP^Sc^ from mammals, PrP is proteinase-sensitive in flies. Thus, wild-type PrP rapidly converts in vivo into a neurotoxic, protease-sensitive isoform distinct from prototypical PrP^Sc^. Next, we investigated the role of molecular chaperones in PrP misfolding in vivo. Remarkably, Hsp70 prevents the accumulation of PrP^Sc^-like conformers and protects against PrP-dependent neurodegeneration. This protective activity involves the direct interaction between Hsp70 and PrP, which may occur in active membrane microdomains such as lipid rafts, where we detected Hsp70. These results highlight the ability of wild-type PrP to spontaneously convert in vivo into a protease-sensitive isoform that is neurotoxic, supporting the idea that protease-resistant PrP^Sc^ is not required for pathology. Moreover, we identify a new role for Hsp70 in the accumulation of misfolded PrP. Overall, we provide new insight into the mechanisms of spontaneous accumulation of neurotoxic PrP and uncover the potential therapeutic role of Hsp70 in treating these devastating disorders.

## Author Summary

Creutzfeldt-Jakob disease is a type of dementia caused by the deposition of the prion protein in the brain. This disorder belongs to a unique class of degenerative diseases that includes mad-cow disease in bovine and scrapie in sheep. An abnormal form of the prion protein is not only responsible for the disease in several mammals, but is also an infectious agent that can transmit the disease within or across species. To shed light on how the prion protein changes from its normal to the disease-causing form, we expressed the prion protein from hamster in transgenic flies. We observed that the prion protein progressively converts to the pathological form and induces neuronal loss in the brain. Thus, the prion protein experiences its typical transition from normal to disease-causing form in flies. This behavior gave us the opportunity to investigate whether other proteins can regulate such transition. We found that the stress-related protein Hsp70 prevents the accumulation of abnormal prion protein and prevents neuronal loss. We also determined that Hsp70 directly interacts with the prion protein in specific membrane domains. Overall, our studies provide new insight into the mechanisms that regulate the accumulation of abnormal prion protein. This discovery could have therapeutic applications in treating these devastating disorders.

## Introduction

The prion protein (PrP) appears to be an essential element in the pathogenesis of an incurable class of neurological disorders called transmissible spongiform encephalopathies (TSE) or prion diseases. These protein deposition disorders can present with sporadic, inherited or infectious origins and lead to dementia, motor dysfunction, and inevitably, death [1]. Regardless of the origin of TSE, conversion of the normal cellular prion protein (PrP^C^) into its pathological scrapie isoform (PrP^Sc^) seems to be the fundamental process underlying the pathogenesis of prion diseases [2]. PrP is a membrane-anchored glycoprotein highly enriched in the brain with a unique ability to undergo conformational changes. PrP^Sc^ can be distinguished from PrP^C^ by its partial resistance to heat, denaturing agents and protease digestion, its insolubility in non-ionic detergents, and its fibrillar aggregation [2]. Moreover, deposition of PrP^Sc^ in the brain is associated with cerebral damage, including spongiform degeneration and neuronal loss. According to the “protein-only” hypothesis, PrP^Sc^ transmits the disease by propagating its abnormal conformation using PrP^C^ as a substrate by autocatalytic mechanisms [2],[3]. It is not clear, though, what other proteins or cellular components are critical for PrP conversion.

The unique infectious aspects of prion diseases have received substantial attention due to the scare of the “mad cow’ epidemics of the 1980's. However, the sporadic disease accounts for 80–85% of all prion disorders in humans [4]. In patients with sporadic Creutzfeldt-Jakob disease (CJD), wild type PrP^C^ converts into typical protease-resistant PrP^Sc^ by mechanisms largely unknown. It has been accepted, though, that intrinsic biochemical properties encoded into the amino acid sequence of PrP are the basis for its conformational changes. Indeed, transgenic mice overexpressing wild type PrP display neuronal loss, astroglyosis, and PrP deposition [5],[6]. Although the role of PrP^Sc^ in transmission has been thoroughly documented, it is not clear whether PrP^Sc^ is the neurotoxic isoform of PrP. PrP conformers that do not share all the biochemical properties of PrP^Sc^ may be responsible for neuropathology in TSE [7],[8]. But the biochemical isolation of this neurotoxic conformer, referred to as PrP*, PrP^toxic^ or PrP^L^ (lethal), has been challenging, so far.

Given the interest in the infectious aspects of prions, the elucidation of the cellular mechanisms involved in spontaneous PrP misfolding and PrP-dependent neurotoxicity has progressed at a slower pace. Recent studies suggest that both endoplasmic reticulum (ER) and cellular stress may play an important role in prion diseases [9]. In fact, PrP misfolding can induce ER stress, which in turn triggers a mechanism of defense characterized by the activation of the unfolded protein response (UPR) and the upregulation of various molecular chaperones. For instance, the ER chaperones Grp58, Grp78/BiP and Grp94, and the heat shock protein Hsp70 are upregulated in the brain of patients affected with CJD and animals infected with scrapie [10]–[12]. However, the protective activity of these molecular chaperones against PrP neurodegeneration has not been assessed in vivo.

In this paper, we have focused on three relevant aspects of PrP biology: Can wild type PrP spontaneously convert in vivo? What is the nature of the neurotoxic PrP species? And, do molecular chaperones play a role in PrP misfolding and aggregation? To answer these questions we expressed wild type PrP from Syrian Golden Hamster (HaPrP) in transgenic flies. The initial isolation of PrP came from hamsters [13] and much knowledge about the biochemical and structural properties of hamster PrP^Sc^ has accumulated over the last 20 years. Since flies do not posses a PrP orthologue, this is a good host system to understand the consequences of expressing mammalian PrP in a genetically tractable model. However, modeling prion diseases in flies has proved challenging [14],[15]. We report here that wild type PrP expressed in *Drosophila* neurons progressively misfolds, acquires biochemical features of PrP^Sc^ and induces spongiform degeneration of brain neurons. Remarkably, the molecular chaperone Hsp70 directly interacts with PrP, prevents the accumulation of misfolded isoforms and reduces its neurotoxicity in neurons of the fly brain. These results suggest that Hsp70 upregulation might be of therapeutic interest in prion diseases.

## Results

### Expression of Mammalian PrP in Transgenic Flies

**Figure 1:**
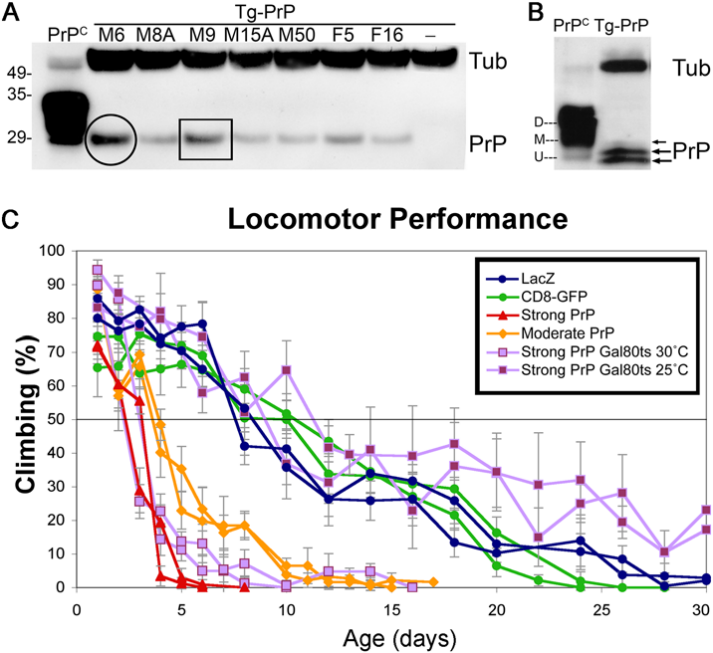
Expression of mammalian PrP and neural dysfunction. (A) Brain expression of high (circled), moderate (box) and weak wild type HaPrP lines (Tg-PrP) in a 4–12% agarose gel (OK107-Gal4/PrP) using the 3F4 anti-HaPrP antibody. A sample of PrP^C^ from hamster brain is shown on the left lane as control. Negative control flies (-) were included and Tubulin (Tub) was used as loading control. (B) In a 15% gel, three bands of Tg-PrP (OK107-Gal4/PrP-M6) are detected corresponding to the Un-, Mono- and Di-glycosylated forms of PrP^C^ from a hamster brain extract. (C) Climbing ability of adult males expressing LacZ (control, blue circles), CD8-GFP (control, green circles), PrP-M6 (red triangles), PrP-M9 (orange diamonds) and PrP-M6; Gal80^TS^ (purple squares) in motor neurons under the control of BG380-Gal4. The strong line PrP-M6 induces early locomotor dysfunction, while the moderate PrP-M9 induces a slightly delayed phenotype measured at 50% climbing. Induction of PrP expression in newly eclosed flies by turning Gal80^TS^ off at 30°C also induces locomotor dysfunction in four days, while siblings placed at 25°C perform as controls.

We created transgenic flies expressing wild type HaPrP, identified strong, moderate and weak HaPrP lines in western blot (Figure 1A), and confirmed that observation by quantitative RT-PCR (Figure S1A). Fly-expressed PrP (Tg-PrP) migrated in a compact band of 28 KDa, unlike control PrP^C^ from a healthy hamster (Figure 1A). PrP^C^ typically produces three distinct bands (35–28 kDa) due to the presence of two facultative *N*-glycosylation sites that yield di-, mono- and unglycosylated PrP fractions (Figure 1A, B). It is well known that glycosylation in flies involves the addition of very small sugar chains and single sugars [16]. Running the fly brain extracts in a high-density gel allowed the separation of three bands, although the higher band showed weaker intensity (Figure 1B). Thus, albeit with slight differences in the glycosylation pattern, PrP is normally processed in *Drosophila*.

### PrP Induces Locomotor Dysfunction and Shortened Lifespan in Flies

To investigate whether wild type PrP expression could cause neuronal dysfunction in flies, we expressed strong and moderate PrP transgenes or control transgenes in motor neurons. As shown in Figure 1C, control males expressing membrane-bound CD8-GFP or cytoplasmic LacZ performed well in climbing assays over 10 days and stopped climbing at around day 25. Interestingly, the strong PrP line triggered a severe locomotor dysfunction only three days after eclosion (measured at 50% of climbing ability) and by day 6 no climbing ability was registered (Figure 1C). We observed a similar result in males expressing the moderate PrP line in motor neurons, although the locomotor dysfunction occurred at day 4 (50% of climbing ability) and the fast decline continued until day 10. Since these flies exhibited an early locomotor dysfunction, we wondered if PrP was affecting motor neuron development. To answer this question, we initiated PrP expression in young adult flies also carrying a temperature-sensitive allele of the Gal4 inhibitor Gal80 [17], thus, preventing PrP expression in developing motor neurons. A temperature shift to the restrictive temperature (Gal80 inactive) in newly eclosed flies (day 1) initiated PrP transcription in mature neurons, which also led to a fast locomotor dysfunction (Figure 1C). In contrast, sibling flies raised at the permissive temperature (Gal80 active, no PrP expression) behaved as control flies. Hence, PrP induces rapid neurotoxicity in motor neurons, and this early neuronal dysfunction is not caused by neurodevelopmental defects.

The progressive neurotoxicity of wild type HaPrP in motor neurons seemed to disagree with a previous report in which wild type PrP from mouse (mPrP) did not induce neurodegeneration [18]. However, the authors clarified later that they had compared a weak wild type mPrP line with a strong mutant mPrP line (*Correction*, *J. Neurosci.*, 2008, Vol. 28). To determine the ability of wild type mPrP to induce degenerative phenotypes, we tested mPrP and HaPrP lines in the same conditions. First, we determined the relative expression levels of the mPrP and HaPrP transcripts by quantitative RT-PCR. We identified mPrP lines expressing slightly lower levels (P1) and twice as much (J1) than our strong HaPrP line (Figure S1A). These mPrP lines induced a strong locomotor dysfunction similar to the defects caused by HaPrP (Figure S1A). While the P1 line showed a slightly delayed locomotor dysfunction compared to HaPrP (at 50% climbing), the J1 line showed a more aggressive phenotype. Therefore, the onset and progression of the locomotor dysfunction correlated with the expression levels of wild type mPrP. These results support the ability of wild type PrP from both mouse and hamster to induce neurotoxic effects.

### Spongiform Degeneration in PrP Flies

**Figure 2:**
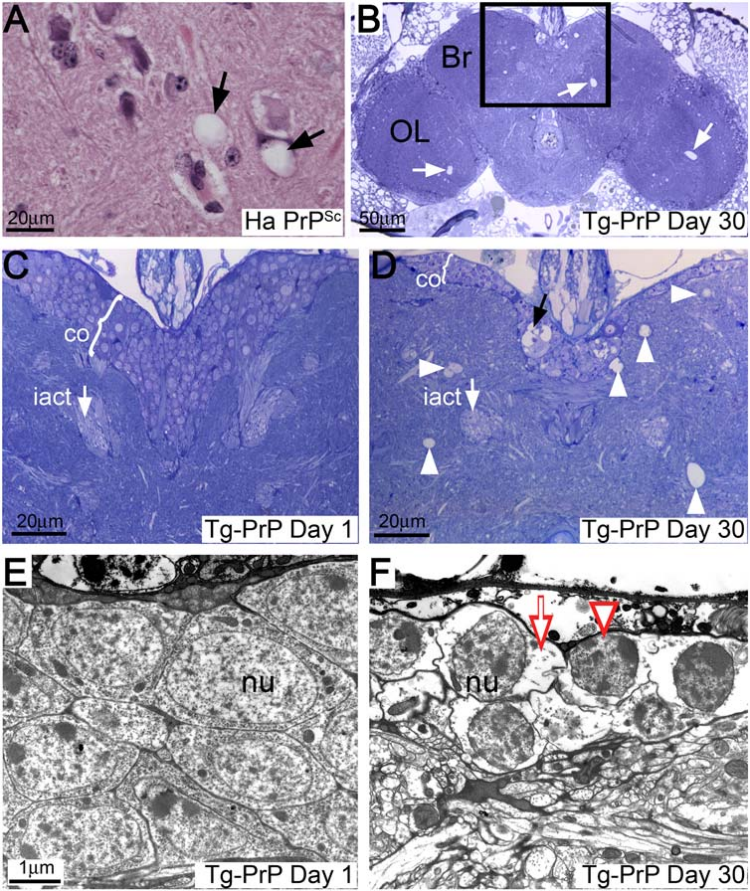
PrP induces spongiform degeneration of brain neurons. (A) The brain of a scrapie-infected hamster (Ha PrP^Sc^) contains vacuolated neurons in the medulla (arrows). (B–D) The brain of a 1 day-old Tg-PrP fly (da-Gal4/PrP-M6) displays a well-preserved neuropile and a thick layer of cells in the cortex (co) (C). In contrast, the brain of a 30 day-old flies contain abundant vacuoles in the brain (Br) and the optic lobes (OL) (B, arrows). The boxed area (magnified in D) shows prominent vacuolar degeneration of the neuropile (arrowheads) and the cortex (black arrow), and the cortex is thinner. The inner antennocerebral tract (iact, white arrow in C and D) is shown as a landmark for section depth. (E and F) Electron micrographs of brain neurons from 1 (E) or 40 day-old (F) Tg-PrP flies. 1 day-old flies exhibit normal cellular morphology (nu = nucleus), but older flies show vacuolated cytoplasm (arrow), nuclear condensation (arrowhead) and abnormal membrane folds.

Spongiform degeneration is the neuropathological hallmark of TSE. In the brain of scrapie-infected hamsters, spongiosis is the consequence of vacuolation of cell bodies and processes (Figure 2A). To investigate the ability of wild type PrP to induce vacuolar pathology in transgenic flies, we expressed HaPrP in all brain neurons and incubated these flies for 1 or 30 days. Young Tg-PrP flies showed well-preserved architecture of the neuropile and the cortex, which contains the cell bodies of the brain neurons (Figure 2C). In contrast, 30 day-old flies displayed large holes in the brain and the optic lobes (Figure 2B). Large and small vacuoles localized to both the neuropile and the cortex (Figure 2D). Additionally, the cortex is much thinner in the older flies, suggesting that a significant neuronal loss occurred. To document the vacuolar pathology at a subcellular level, we performed ultrastructural analysis of the fly brains. While young Tg-PrP flies exhibited normal cellular organization (Figure 2E), older Tg-PrP flies clearly showed cytosolic vacuolation, nuclear condensation and abnormal membrane folding (Figure 2F). Therefore, accumulation of wild type PrP for 30 days leads to severe spongiform vacuolar degeneration of *Drosophila* brain neurons, a hallmark of prion neuropathology.

### PrP Undergoes Progressive Misfolding

**Figure 3:**
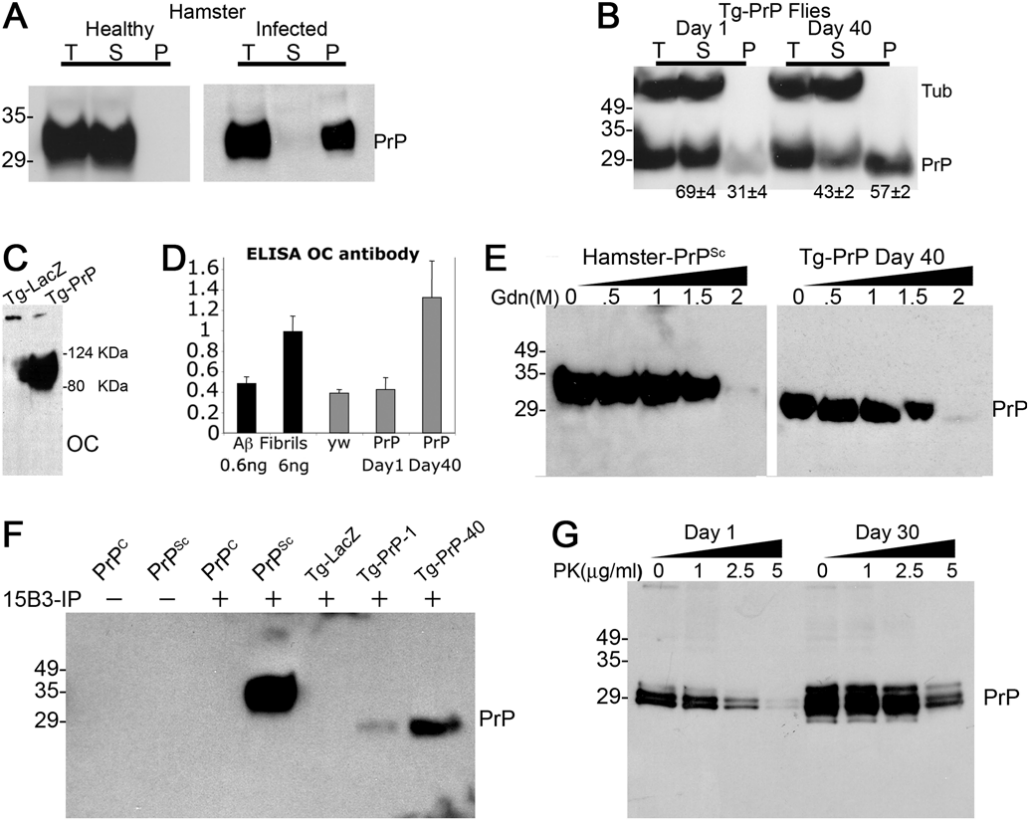
Spontaneous accumulation of misfolded PrP. (A and B) Insolubility of PrP^Sc^ and Tg-PrP. (A) PrP^C^ from a healthy hamster is only present in the sarkosyl/NaPTA soluble fraction (S), while PrP^Sc^ from a scrapie-infected hamster is only detected in the pellet (P). A total (T) equivalent of the extract is also shown. (B) Young Tg-PrP flies produce mostly soluble PrP (69% of total PrP), whereas most PrP becomes insoluble in 40 day-old flies (57% of total PrP). Tubulin (Tub) is never detected in the insoluble pellet. All the flies used in this figure were OK107-Gal4/PrP-M6. (C and D) Fibrillar conformation of Tg-PrP. (C) Fibrillar conformations were detected in western blot with the OC antibody in head extracts from 40 day-old Tg-PrP flies, but not in Tg-LacZ flies, as a high molecular weight signal. (D) Quantitation of OC immuno reactivity in ELISA. Brain extracts from old Tg-PrP flies produce high signal compared to young Tg-PrP flies and control (yw) flies. Reactivity against amyloid-β fibrils is also shown as control for OC activity. (E) Resistance to denaturing agents. Brain extracts from scrapie-infected hamster (left) and 40 day-old Tg-PrP flies (right) were treated with a gradient of guanidine thiocyanate (Gdn) and the insoluble PrP was detected by western blot. 2 M Gdn is required to completely solubilize both PrP^Sc^ and Tg-PrP. (F) Tg-PrP acquires a PrP^Sc^-like conformation. The 15B3 conformational antibody immunoprecipitates PrP^Sc^, but not PrP^C^, from hamster brain extracts. Tg-PrP flies also accumulate 15B3-reactive conformers, particularly in older flies. 40 day-old Tg-LacZ flies (control) rendered no signal. (G) Tg-PrP is PK-sensitive. Brain extracts from Tg-PrP flies (da-Gal4/PrP-M6) treated with a mild PK gradient yielded no PK-resistant core (shift). However, Tg-PrP from older flies shows a relative resistance to a mild PK gradient compared to young Tg-PrP flies. Additionally, older flies accumulate an extra band that could be a degradation product of Tg-PrP.

A classical finding in TSE is the misfolding of PrP^C^ into new conformers that are insoluble in non-ionic detergents [2]. To determine if the neurodegeneration described in the fly brain correlated with PrP misfolding, we assessed PrP solubility in mammalian and fly brains. For this, we treated hamster and fly brain extracts with a non-ionic detergent (sarkosyl) and Na-phosphotungstate (NaPTA), a reagent that promotes specific precipitation of PrP^Sc^ [19]. Then, the soluble and insoluble fractions were resolved by western blot. As expected, control PrP^C^ from a healthy hamster was only detected in the sarkosyl/NaPTA soluble fraction, while PrP^Sc^ from a scrapie-infected hamster accumulated in the insoluble fraction (Figure 3A). We next analyzed the biochemical properties of Tg-PrP expressed in a subset of brain neurons (the mushroom bodies, see Figure 5C) in young flies and flies aged for 40 days. Western blots showed that Tg-PrP was mostly soluble in young flies, while Tg-PrP showed marked insolubility in 40-day old flies (Figure 3B). To determine the specificity of these changes in PrP properties, we examined the solubility of another exogenous protein, a cytosolic form of bacterial β-Galactosidase. β-Galactosidase demonstrated complete solubility in both young and older flies (Figure S2). This result argues against the possibility that the insolubility of PrP in aged flies could be due to the deterioration of cellular homeostasis systems or to the overexpression of high, non-physiological levels of any exogenous protein.

Next, we confirmed the fibrillar state of Tg-PrP by using a conformation-dependent antibody (OC) that detects common epitopes in fibrils of different types of amyloids [20]. To preserve the OC-reactive epitopes, we generated brain homogenates without detergents and, then, ran the samples by denaturing PAGE. Immuno-detection with the OC antibody recognized a high molecular weight band in Tg-PrP flies, but not in control flies (Figure 3C), consistent with the range of fibrillar structures recognized by this fibril-specific antibody [20]. We also quantified OC reactivity by ELISA and found that aged Tg-PrP flies produced a three-fold higher signal compared to younger flies and control flies (Figure 3D). In summary, the biochemical study of Tg-PrP indicates that wild type PrP progressively misfolds and forms insoluble, fibrillar conformers that could be responsible for the neurodegenerative phenotype.

### PrP Acquires Biologically Relevant Conformations in *Drosophila*

PrP exhibits an unusual flexibility that allows it to acquire a number of conformations both in vivo and in vitro [21]. To better understand the conformation accumulated in flies, we compared the resistance of Tg-PrP and PrP^Sc^ from hamster to denaturing agents. PrP^Sc^ is highly resistant to heat, urea and guanidine thiocyanate; however, high concentration of chemical denaturants can destabilize the PrP^Sc^ conformation, rendering it soluble. We subjected extracts from scrapie-infected hamsters and heads from old flies to a gradient of guanidine thiocyanate and then tested for PrP solubility. Both PrP^Sc^ from hamster and Tg-PrP from old flies exhibited remarkable resistance to high concentrations of guanidinium (up to 1.5 M) (Figure 3E). Interestingly, both PrP samples were solubilized at 2 M guanidinium. The comparable guanidinium sensitivity of PrP^Sc^ and Tg-PrP suggests that the conformation acquired by PrP in transgenic flies may be similar to that of PrP^Sc^ from infected hamsters.

To further characterize the conformation of fly-expressed PrP, we used the 15B3 antibody [22], which discriminates normal (PrP^C^) from disease-specific (PrP^Sc^) conformations in bovine, sheep, rodents and human CJD (Figure 3F). Then, we performed immunoprecipitation with 15B3 in either control (Tg-LacZ) or Tg-PrP flies. 15B3 did not cross-react with brain extract from Tg-LacZ flies, but recognized a small amount of PrP^Sc^-like conformers in young Tg-PrP flies. Interestingly, 40-day old Tg-PrP flies produced a much larger amount of immunoprecipitated 15B3 conformers (Figure 3F). Thus, Tg-PrP flies spontaneously and progressively accumulated biologically relevant conformations that share specific epitopes with PrP^Sc^.

### Misfolded PrP Is PK Sensitive in *Drosophila*

One of the most typical features of infectious PrP^Sc^ is its resistance to high concentrations of proteinase K (PK) and the production of a protease resistant core of smaller size (PrP^27–30^) [2]. We subjected brain extracts from PrP transgenic flies to mild PK digestions and, then, we resolved the products by western blot using a small pore membrane (.2 µm) for increased sensibility. In these conditions we could observe several differences between the extracts from young and old flies, but no PK-resistant core (shift) was detected. Still, Tg-PrP showed a relative increase in PK resistance in the older flies, consistent with the accumulation of misfolded PrP (Figure 3G). Older flies also accumulated a new band in the non-digested sample just below the lowest band expected for Tg-PrP, suggesting that this was a degradation product accumulated over time. Thus, although transgenic flies did not produce PrP conformers with the complete biochemical properties of PrP^Sc^, our results are consistent with other PrP conformations that might be relevant in disease [8].

### Human Hsp70 Regulates PrP Conformation

**Figure 4:**
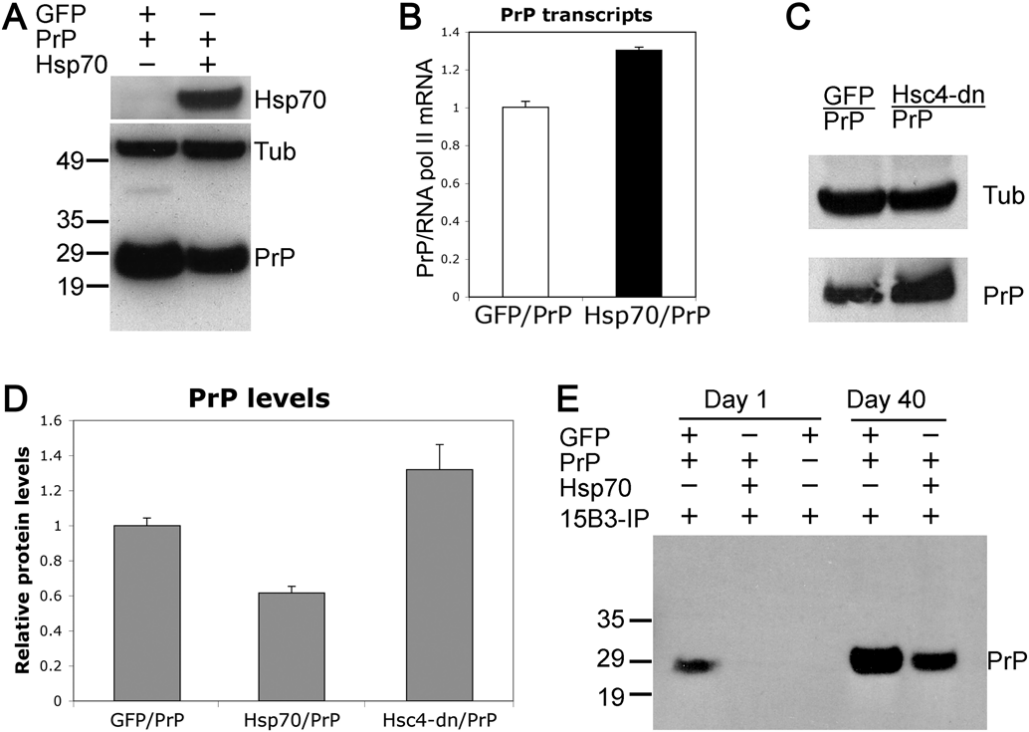
Hsp70 prevents PrP misfolding. (A–D) Hsp70 modulates PrP dynamics. (A) 1 day-old flies co-expressing Hsp70 and PrP (OK107-Gal4/h-Hsp70/PrP-M6) accumulate less total PrP than controls (OK107-Gal4/CD8-GFP/PrP-M6). (B) Quantitative RT-PCR indicates that the levels of PrP transcripts are not reduced by Hsp70. (C) 1 day-old flies co-expressing Hsc4-dn and PrP (OK107/Hsc4-dn/PrP-M6) accumulate more total PrP than controls in western blot. (D) Quantitation of PrP in western blot indicates that Hsp70 reduces total PrP by 40%, while Hsc4-dn increases PrP by 35% (p<0.001). Densitometry was averaged from three independent experiments and normalized against Tub. (E) Hsp70 prevents the accumulation of PrP^Sc^-like conformers. 1 day-old control GFP/PrP flies accumulate a small amount of 15B3 immunoreactivity, but Hsp70 completely eliminates 15B3-reactive PrP isoforms. 40 day-old Hsp70/PrP flies show a dramatic reduction in 15B3 signal compared with old GFP/PrP flies.

Among the chaperone proteins Hsp70 has the exceptional ability to correct the misfolding of several amyloidogenic proteins involved in neurodegenerative diseases [23],[24]. However, very little is known about its potential role in extracellular amyloid diseases such as TSE. To test the ability of Hsp70 to functionally interact with PrP, we took advantage of the construct expressing human Hsp70 shown to rescue polyglutamine and α-Synuclein neurotoxicity in flies [25],[26]. We overexpressed PrP and Hsp70 in brain neurons and, then, investigated whether Hsp70 elicits changes on the conformation, turnover and/or stability of Tg-PrP. Surprisingly, Hsp70/PrP flies accumulated less total PrP than control flies GFP/PrP (Figure 4A). Densitometry of three independent experiments indicated that flies co-expressing Hsp70 accumulated 40% less PrP (Figure 4D). Moreover, this reduction in the levels of PrP was exerted post-translationally since Hsp70 did not interfere with the production or stability of PrP transcripts (Figure 4B). Consistent with this result, flies co-expressing PrP and a dominant negative variant of constitutive Hsp70 (Heat shock cognate 4, Hsc4-dn) accumulated 35% more PrP (Figure 4C, D), supporting a role for Hsp70 in PrP biology. Then, we examined whether Hsp70 promoted the elimination of specific PrP conformations using the 15B3 conformational antibody. While young GFP/PrP (control) flies accumulated a small amount of 15B3 immunoreactive species, young Hsp70/PrP flies did not accumulate 15B3-positive epitopes (Figure 4E). Moreover, older Hsp70/PrP flies accumulated much lower levels of 15B3 immunoreactive species than the control GFP/PrP flies (Figure 4E). These observations suggest that Hsp70 prevents the accumulation and/or promotes the degradation of specific PrP conformers, and support a role for Hsp70 in regulating PrP conformation.

### Human Hsp70 Protects against PrP Neurotoxicity

**Figure 5:**
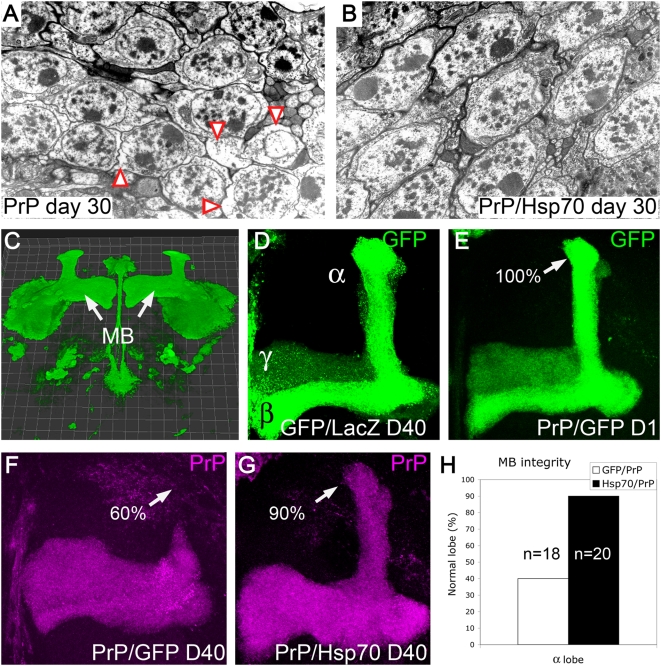
Hsp70 protects against PrP neurotoxicity. (A and B) Ultrathin sections of 30-day old PrP flies (da-Gal/PrP-M9) contain a mix of cells showing either vacuolated (red arrows) or preserved cytoplasm (A). However, co-expression of Hsp70 (da-Gal4/h-Hsp70/PrP-M9) leads to a homogeneously preserved cellular morphology (B). (C–H) Hsp70 protects the mushroom body projections. (C) 3-D reconstruction of the mushroom body (MB) system in the fly brain (OK107-Gal4/CD8-GFP/LacZ). (D) Detail of the α, β and γ lobes showing normal mushroom body morphology in a 40-day old control fly (OK107-Gal4/CD8-GFP/LacZ). (E) 1-day old flies expressing GFP and PrP (OK107-Gal4/CD8-GFP/PrP-M6) also display normal mushroom body morphology (arrow). (F) However, 60% of 40-day old flies (n = 18) exhibit degenerated α lobes as shown by anti-PrP staining (arrow). In contrast, 90% of 40-day old flies co-expressing Hsp70 and PrP (OK107/h-Hsp70/PrP-M6, n = 20) display normal α lobe morphology (G, arrow). (H) Representation of the fraction of mushroom bodies with normal morphology.

We followed these experiments by assessing the ability of Hsp70 to protect against PrP-dependent neurotoxicity. First, we wondered if Hsp70 could protect against the vacuolar degeneration of brain neurons induced by Tg-PrP. For this, we created flies that exhibited an intermediate spongiform phenotype by using a moderate PrP line. The rationale for this moderate phenotype was to provide sensitive conditions to detect Hsp70 neuroprotection. Flies expressing moderate PrP levels in all neurons showed a mixed phenotype at day 30, where 44% of cells were undergoing vacuolar changes, while the rest exhibited preserved cytoplasm (Figure 5A). The nuclei of most of these neurons were condensed, suggesting that most cells were undergoing apoptosis. In contrast, flies co-expressing Hsp70 and PrP exhibited very few vacuolated cells (7%) and their nuclei were normal, suggesting that Hsp70 prevents spongiform degeneration and cell death (Figure 5B).

Next, we analyzed the mushroom bodies, which are the dorsal (α lobe) and medial (β and γ lobes) projections of the Kenyon cells (Figure 5C, D). As expected, young flies co-expressing GFP with a strong PrP line and 40 day-old control flies showed normal mushroom body morphology (Figure 5D, E). However, 40 day-old GFP/PrP flies displayed prominent degeneration of α lobes (Figure 5F, H). Remarkably, flies co-expressing Hsp70 and PrP demonstrated robust protection of mushroom body projections (Figure 5G, H). These results further confirmed the ability of Hsp70 to prevent the progressive degeneration of neuronal structures.

**Figure 6:**
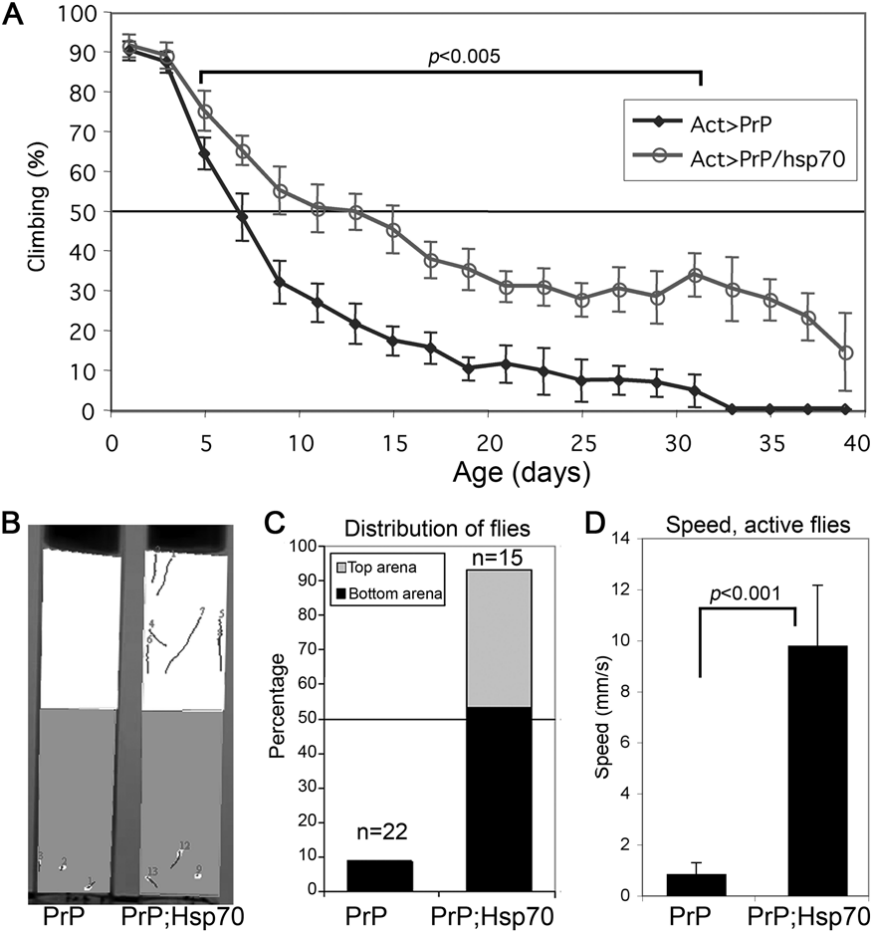
Hsp70 prevents the locomotor dysfunction induced by PrP. (A) Flies co-expressing Hsp70 and PrP (Act-Gal4/PrP-M9/h-Hsp70) perform better in climbing assays than flies expressing only PrP (Act-Gal4/PrP-M9). From day 5 to day 31, the climbing ability of PrP and Hsp70/PrP flies was significantly different. (B–D) Software-assisted analysis of fly movement. (B) Flies entering two identical arenas (top and bottom halves) in 10 sec are detected using GroupScan. (C) 40% of 20 day-old Hsp70/PrP flies occupy the top arena, while another 55% occupy the bottom arena. Only 5% of these flies are not detected by the software because they do not enter either arena. In contrast, 8% of PrP-only flies enter the bottom arena but none climb to the top arena. (D) Average speed measurements in arenas spanning the entire vial demonstrate the dramatic difference between PrP and Hsp70/PrP flies.

Finally, we tested the protective activity of Hsp70 in functional assays. For this, we established a moderate locomotor phenotype by inducing a ubiquitous, albeit weak, expression of PrP. Under these conditions, the flies expressing only PrP displayed a steady decline in climbing ability over 20 days, with a 50% climbing activity at day 7 (Figure 6A). Flies expressing PrP and Hsp70 also showed a steady, but less pronounced decline, with a 50% climbing activity at day 13 (Figure 6A). From day 5 to day 31 the differences in climbing ability were highly significant. We further characterized the protective activity of Hsp70 by analyzing the movement of these flies at day 20. Groupscan can identify and track multiple flies that enter a custom arena, records the movement of all the flies in the arena and produces parameters characteristic of each population (Figure 6B). To document the protective activity of Hsp70, we measured the number of flies that occupied the top and bottom halves of the vial after 8 seconds. Flies expressing PrP alone were never detected in the top arena, while only three out of 22 flies entered the bottom arena. These observations indicated that most of the flies stayed in the floor of the vial because they could not climb vertically (Figure 6B, C). In contrast, 40% of the PrP/Hsp70 flies occupied the top arena and only one fly out of 15 stayed in the floor of the vial (Figure 6B, C). To determine if the speed of these flies could be a more sensitive parameter to describe the protective activity of Hsp70, we measured the average speed per fly in arenas that occupy the whole vial. PrP/Hsp70 flies performed much better than PrP-only flies, exhibiting a speed ten times higher (Figure 6D). Fly distribution and speed clearly illustrated the improved locomotor ability of the flies co-expressing Hsp70 and PrP. Overall, the protective effect of Hsp70 against the locomotor dysfunction, the spongiform vacuolation and the axonal degeneration suggests that Hsp70 effectively protects both neural morphology and activity against PrP neurotoxicity in vivo.

### Hsp70 Directly Interacts with PrP in Membranous Domains

**Figure 7:**
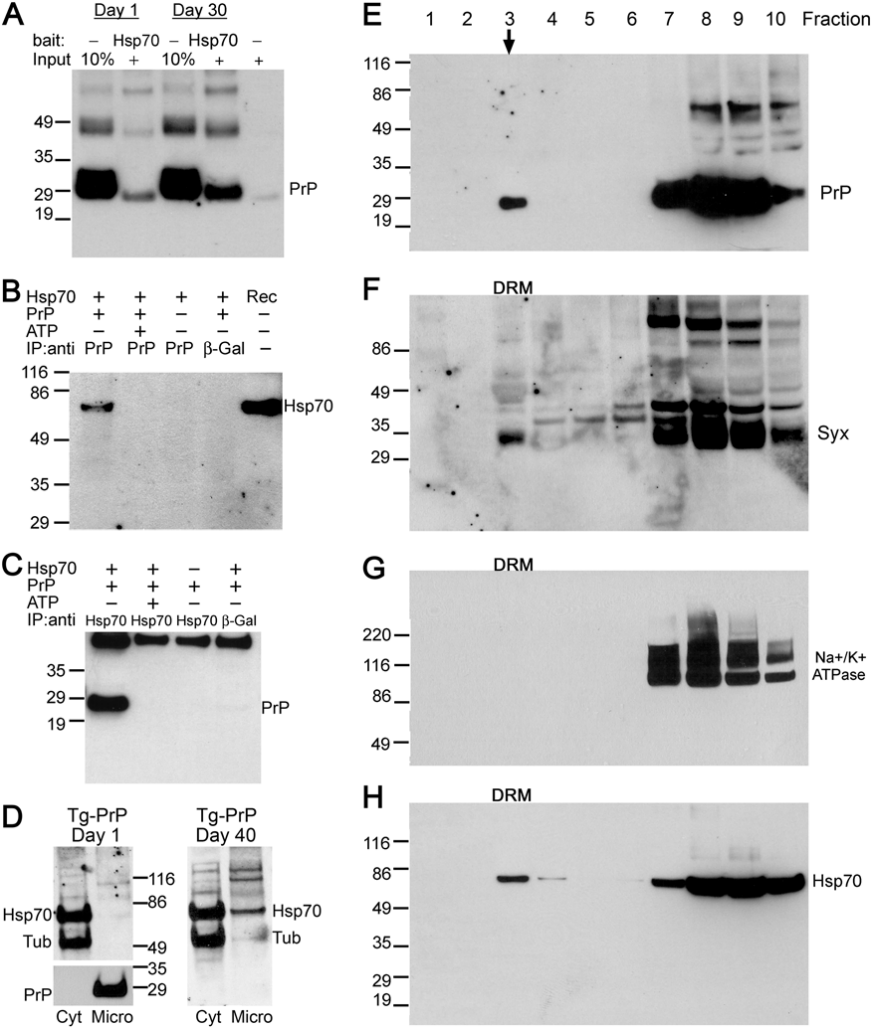
Hsp70 interacts with PrP and is present in lipid rafts. (A) Pull-down assay showing PrP interaction with h-Hsp70. Tg-PrP from young and old flies (da-Gal4/PrP-M6) binds immobilized h-Hsp70. However, Tg-PrP from older flies showed higher affinity for Hsp70. Aliquots of the homogenates were also loaded directly on the gel (10% input). Lane 5 is a control pull-down with beads without bait (h-Hsp70). (B and C) Co-immunoprecipitation of PrP and Hsp70. (B) Brain extracts from Hsp70/PrP flies (da-Gal4/h-Hsp70/PrP-M6) produced Hsp70 immunoreactivity when they were immunoprecipitated with anti-PrP (lane 1), but not with an unrelated antibody (anti-β-Galactosidase, lane 4). The Hsp70 immunoprecipitation was reversed with the addition of ATP to the mix (lane 2). Hsp70-only flies (da-Gal4/h-Hsp70) did not produce signal when immunoprecipitated with the PrP antibody (lane 3). As control, we show recombinant Hsp70 (Rec) in lane 5. (C) In the reverse experiment, PrP is detected when extracts are immunoprecipitated with anti-Hsp70 antibody (lane 1) and this interaction is reverted by ATP (lane 2). Flies expressing only PrP (da-Gal4/PrP-M6, lane 3) or beads with the anti-β-Galactosidase antibody (lane 4) did not bring down PrP. (D) PrP and Hsp70 in microsomes. Separation of cytoplasmic (Cyt) and microsomal (Micro) fractions from brain extracts (da-Gal4/PrP-M6) shows PrP distribution in membranous vesicles (left). Endogenous Hsp70 accumulates exclusively in the cytoplasm in 1 day-old PrP flies, but it is also detected in the microsomal fraction in 40 day-old flies (right). (E–H) PrP and Hsp70 in lipid rafts. (E) PrP is present in the floating fraction number 3 (arrow) collected from an Optiprep gradient in flies da-Gal4/h-Hsp70/PrP-M6. The bottom fractions of the gradient also contain PrP. As control for the separation of lipid rafts, Syntaxin (Syx) also localizes to fraction 3 containing detergent resistant membranes (DRM) (F), while the Na+/K+ ATPase transmembrane pump is excluded from DRM (G). Hsp70 also floats to DRM in flies co-expressing PrP and Hsp70 (H).

To better understand how Hsp70 exerts its chaperone activity on PrP, we evaluated the possibility that Hsp70 could interact directly with PrP. For this, we performed pull-down and co-immunoprecipitation assays in flies co-expressing PrP and Hsp70. For the pull-down we prepared active His-Hsp70 coated beads in a spin column and tested the binding of Tg-PrP from brain homogenates of young and old flies. Then, we resolved the interacting fraction in western blot and assessed PrP Immunoreactivity. Tg-PrP from both young and old flies interacted with Hsp70 in the column, but the amount of PrP recovered from older flies was several fold higher (Figure 7A). This result confirmed the interaction of Hsp70 with specific PrP conformers that accumulate in older flies, possibly misfolded PrP. To determine the biological relevance of this in vitro interaction, we next performed co-immunoprecipitation assays. As hypothesized, Hsp70 co-immunoprecipitated with PrP using anti-PrP coated beads in brain extracts from older Hsp70/PrP flies, but was not detected in extracts from flies expressing only Hsp70 (Figure 7B). In addition, Hsp70 was not precipitated when beads were coated with an unrelated antibody (anti-β-Galactosidase) (Figure 7B). To test the physiological relevance of this interaction, we added ATP to the binding reaction to induce Hsp70 cycling, resulting in the release of its substrate. Interestingly, Hsp70 was not detected in the eluted fraction in the presence of ATP (Figure 7B). Then, we confirmed the interaction between Hsp70 and PrP by performing the reverse immunoprecipitation with anti-Hsp70 coated beads. In this case, PrP immunoprecipitated with Hsp70 in flies co-expressing both transgenes, but not in flies expressing only PrP or in beads coated with a control antibody (Figure 7C). Similarly, when ATP was added to the reaction, the binding of Hsp70 and PrP was reversed (Figure 7C). Combined, these results strongly suggest that Hsp70 exerts its protective activity by direct interaction with PrP.

These intriguing results presented a clear problem: where does the interaction between Hsp70 and PrP take place? Hsp70 is the major cytosolic chaperone, while PrP is a secreted glycoprotein attached to the extracellular membrane. Hence, the chances for these two proteins to physically interact seemed limited. Therefore, we wondered if we could identify the subcellular domain where Hsp70 and PrP interact. Recent reports show that Hsp70 exhibits a remarkable ability to translocate to different cellular compartments, including the extracellular space [27]. To determine if we could detect Hsp70 in membranous domains, we first separated the cytosolic and microsomal fractions. Microsomes contain membrane vesicles from all cellular compartments, including ER, Golgi, secretory and recycling vesicles, and plasma membrane. Since PrP is processed in the ER and Golgi and is anchored to the membrane, it accumulated solely in the microsomal fraction (Figure 7D). As expected, endogenous Hsp70 localized in the cytosolic fraction in young Tg-PrP flies (Figure 7D). However, when we analyzed the distribution of Hsp70 in older Tg-PrP flies, a significant amount of endogenous Hsp70 was present in the microsomal fraction. This observation suggests that Hsp70 can translocate to membranous domains in response to the accumulation of specific PrP conformers in aged animals.

A relevant cellular microdomain for PrP biology is the lipid raft or detergent-resistant membrane (DRM), an specialized plasma membrane domain involved in critical cellular functions, such as trafficking, signalling and protein sorting. Moreover, the lipid raft has been proposed as the site for PrP conversion [28]. To determine if Hsp70 is present in this key plasma membrane domain, we performed a fractionation of fly brain extracts in Optiprep gradients containing 1% Triton X-100. Under these conditions, lipid rafts float to the top fractions, where they are visible as a white fatty material. We confirmed, first, the localization of PrP to the lipid raft, corresponding to floating fraction 3 (Figure 7E). PrP is also detected in the bottom fractions, but its presence in the specialized membranes of fraction 3 is highly relevant. The quality of the separation was assessed by detecting the synaptic protein Syntaxin (a lipid raft marker) in fraction 3 (Figure 7F) and by the absence of the Na+/K+ ATPase, a transmembrane ion pump enriched in non-lipid raft membranes (Figure 7G). Interestingly, a significant amount of Hsp70 was also present in fraction 3 (Figure 7H). Thus, Hsp70 and PrP co-localize in a biologically active microdomain of the membrane that is also the site for PrP conversion, where Hsp70 can interfere with PrP misfolding and promote PrP degradation.

## Discussion

### Spontaneous Conversion of Wild Type PrP In Vivo

Considerable attention has been devoted in the last 25 years to define the chemical nature of prions and their transmissibility. However, less is known about the cellular mechanisms that participate in PrP misfolding, how prions actually damage the central nervous system and how this process can be prevented. To understand the mechanisms regulating spontaneous PrP misfolding, we described how the biochemical properties of wild type PrP change over time in transgenic flies. Early on, Tg-PrP is mostly soluble and accumulates very little misfolded conformers. In contrast, Tg-PrP from older flies is mostly insoluble, fibrillar, resistant to high concentrations of guanidinium and is recognized by a PrP^Sc^-specific conformational antibody. These new features suggest that wild type PrP progressively misfolds and accumulates in a conformational state that shares several properties with PrP^Sc^. However, this PrP conformer is protease sensitive, which clearly distinguishes it from prototypical PrP^Sc^. Hence, Tg-PrP acquires a conformation consistent with PrP isoforms previously described in both experimental animals and patients [29],[30]. These PrP*, PrP^toxic^, PrP^L^ or protease-sensitive PrP^Sc^ conformers have been interpreted as PrP^Sc^ byproducts or, alternatively, they could be immature metabolic intermediaries of PrP^Sc^ [8],[29]. A possible factor in the formation of a PrP* conformer in flies may be the short incubations assayed in these animals (30–40 days). Other explanations could include the lack of co-factors necessary to promote the PrP^Sc^ conformation (conversion factor) or the presence of molecules that prevent the formation of PrP^Sc^ (anti-conversion factor). It is not clear, thus, why flies accumulate this specific PrP conformer or whether flies could produce PrP^Sc^ through genetic modification of the cellular environment. These relevant aspects of PrP biology can be further studied in Tg-PrP flies through genetic studies.

### Understanding the Nature of Neurotoxic PrP

We describe here the formation of a neurotoxic PrP conformer that leads to typical spongiform vacuolation of brain neurons. But, what have we learned about the mechanisms of PrP neurotoxicity? Conversion of PrP^C^ to PrP^Sc^ is central to prion pathogenesis because *Prnp* null mice and mice in which PrP expression is knocked-out after infection are resistant to disease [31],[32]. However, increasing evidence argues against the neurotoxicity of PrP^Sc^ because significant pathology and/or clinical dysfunction can develop with little accumulation of protease-resistant PrP^Sc^ in rodent models of TSE [33],[34]. Moreover, a new prion disease in humans has been associated to protease-sensitive PrP [30]. Thus, it is not clear whether specific conformers are associated with neurodegeneration. Our data, though, support the hypothesis that PrP* or PrP^L^ conformers induce deleterious effects by gain-of-function mechanisms since neurotoxicity in flies correlates with the progressive accumulation of novel, protease-sensitive PrP^Sc^-like conformers [8]. We still do not understand how these conformers accumulate in flies. But according to the “templated toxic intermediate” model of J. Collinge, a high rate of conversion of PrP^C^ to PrP^L^ and a low rate of maturation of PrP^L^ to PrP^Sc^ would favor the accumulation of neurotoxic conformers [29]. Transgenic flies seem to lack the maturation phase and, thus, only accumulate PrP^L^, resulting in strong neurotoxicity. These results also suggest that neurotoxic PrP can form independently of the typical PrP^Sc^ pathway and may represent a stable conformer with its own kinetics. Once the PrP^L^ conformers accumulate, they can exert neurotoxicity by sequestration of cellular proteins, inhibition of the cellular clearance machinery (molecular chaperones, the Ubiquitin-Proteasome Complex [UPC]), and/or induction of ER stress and the UPR, among other mechanisms. These deleterious effects of wild type PrP in flies are consistent with the brain and muscle defects observed in transgenic mice that overexpress wild type PrP [5],[6],[35]. Moreover, the ability of wild type PrP to misfold into a neurotoxic conformer fits nicely with the “permissive templating” hypothesis, which proposes that the quantity of the normal protein influences the risk of sporadic diseases, including TSE and Alzheimer and Parkinson's diseases [36].

So, can these new PrP* conformers generated in *Drosophila* be considered prions? Based on the classic definition, which includes transmissibility, PrP* does not share all properties of prions since it is not protease resistant, an important feature for PrP infectivity. However, transmissibility has been achieved with protease sensitive material in some instances [34]. Consequently, some authors propose that prions should be defined based on disease-inducing activity, not on their resistance to protease digestion [33]. A recent report by Chiesa and col. described the spontaneous accumulation of misfolded, neurotoxic PrP in transgenic mice overexpressing wild type PrP [37]. The properties of PrP in these mice is very similar to that described here in transgenic flies expressing wild type PrP. Since brains extracts of these mice were not infectious, it may be safe to assume that Tg-PrP from flies will not be infectious either. However, we will know the answer to this question once our ongoing experiments are finalized.

### Hsp70 Prevents PrP Conformational Changes and Neurotoxicity

Hsp70 is one of the most potent molecular chaperones and has been shown to prevent misfolding of α-Synuclein and expanded polyglutamine proteins in transgenic flies [23],[25]. Hsp70 also prevents neurodegeneration in mouse models of spinocerebellar ataxias and spinal and bulbar muscular atrophy [38],[39]. These protein misfolding disorders are characterized by the presence of nuclear or cytosolic aggregates, where the direct activity of Hsp70 is possible. In contrast, no role has been proposed, so far, for Hsp70 and other cytosolic chaperones in extracellular amyloids such as Amyloid-β and PrP. Probing the protective activity of Hsp70 in PrP-expressing transgenic flies, we found that Hsp70 prevents the accumulation of neurotoxic, PrP^Sc^-like conformers, and involves the direct binding of Hsp70 and PrP. Supporting this idea, the direct interaction of Hsp70 to cytosolic PrP (cytPrP) prevents apoptosis in cultured neurons [40]. Since the physiological relevance of cytPrP is unclear, a key question in PrP biology is whether Hsp70 can interact with the normal membrane-tethered PrP. Under stress conditions Hsp70 can move across membranous structures and into organelles [41]. Indeed, Hsp70 can be released into the extra-cellular space via exosomes [27] and can also pull proteins across membranes [42]. Furthermore, Hsp70 has been detected in lipid rafts in normal cells, a plasma membrane microdomain critical for PrP biology, while stress conditions exacerbate this distribution of Hsp70 [43]. In this study we show that Hsp70 can localize to cellular vesicles (microsomes) and, more specifically, to lipid rafts, providing a physical site for its interaction with PrP. We also present a mechanistic model for the neuroprotective activity of Hsp70 through the interaction with PrP in a key cellular compartment in which PrP misfolding might be occurring. This activity of Hsp70 may prevent or revert PrP conformational changes, while promoting the degradation of misfolded conformers through the UPC. These results agree with and may explain the observation that Hsp70 levels are elevated in patients affected with CJD and in animal models of TSE [10],[11].

It is possible that the Hsp70/PrP interaction is mediated by Hsp70 co-chaperones, such as Hsp40. Hsp40 directly binds substrates and presents them to the catalytic site of Hsp70 [42]. Thus, chaperone complexes that contain Hsp70 could bind PrP and directly modulate PrP conformation, stability and/or degradation in concert with the ubiquitin-proteasome complex. It would be interesting, therefore, to test if other families of chaperones, including the chaperonins (Hsp60's) and the small chaperones (Hsp20's), also regulate PrP misfolding. Regardless of the mechanism mediating Hsp70 protection, this is the first evidence that a molecular chaperone can directly protect against PrP neurotoxicity in vivo.

## Materials and Methods

### Generation of Transgenic Flies

The open reading frame of the Syrian golden hamster *Prnp* gene was isolated by PCR amplification from genomic DNA. EcoRI and NotI restriction sites were included in the primers (5′-GAATTCATCATGGCGAACCTTAGCTACTG-3′ and 5′-GCGGCCGCTCATCCCACCATCAGGAAGATG-3′) to facilitate cloning into the *Drosophila* pUAST vector [44]. The resulting construct (UAS:HaPrP) was injected into *yw* embryos and seven single-insertion lines were created.

### *Drosophila* Stocks and Genetics

UAS flies expressing human Hsp70 (HSPA1L), *Drosophila* Hsc4-dn (HSC4-K71S), the reporter strains UAS:LacZ and UAS:CD8-GFP, and the mushroom body (OK107-Gal4), motor neuron (BG380-Gal4) and ubiquitous (da-Gal4, Act-Gal4) drivers were obtained from the Bloomington Drosophila Stock Center. Two strong mPrP strains were provided by S. Supattapone [18]. Homozygous females for the drivers were crossed with males bearing either HaPrP-M6 (strong) and HaPrP-M9 (moderate) combined with Hsp70, CD8-GFP or LacZ transgenes. To balance Gal4 activity, control (CD8-GFP/LacZ) and experimental (CD8-GFP/PrP and Hsp70/PrP) progenies always carried two UAS transgenes. The crosses and their respective progenies were kept at 28°C unless otherwise indicated.

### Cellular and Histo-Pathological Characterization

Flies expressing PrP throughout the brain under the control of da-GAL4 were collected at 1 and 30 days after eclosion, along with sibling control flies. Plastic embedding was prepared as described [45], then semithin sections were cut at 1 µm and stained with toluidine blue. Ultrahin sections were cut at 70 nm and stained with uranyl acetate (1 h) and lead citrate (15 min). Paraffin brain sections (6 µm) from sick hamsters and H&E staining were performed as described [46]. Whole-mount immunohistochemistry was conducted as described [45] using the anti-HaPrP antibody 3F4 (1∶1,500, Signet) and anti-human Hsp70 antibody (1∶2,000, StressGen). The anti-Mouse-Cy3 (Molecular Probes) and anti-Rabbit-FITC (Sigma) antibodies were used at 1∶600 and images were collected in a LSM510 confocal microscope.

### Locomotor and Longevity Assays

Flies carrying HaPrP-M6, HaPrP-M9, mPrP-P1, mPrP-J1 or control constructs were crossed with the BG380-Gal4 driver and the progeny was subjected to climbing assays [47]. Flies also carrying the transcriptional repressor Gal80^TS^ were raised at 18°C and the adults were placed at either 25°C (no expression) or 30°C (high PrP expression) upon eclosion [17]. For Hsp70 activity, we crossed a milder driver (Act-Gal4) with HaPrP-M9 or HaPrP-M9; h-Hsp70 flies. Briefly, 30 newborn adult males were placed in empty vials and forced to the bottom by firmly tapping against the surface. After 8 seconds, the number of flies that climb above 5 cm was recorded. This was repeated 8 times every 1 or 2 days for 30 days. Climbing ability was plotted as a function of age. For software-assisted analysis, we recorded the climbing for 10 seconds and the videos were analyzed with Groupscan (Cleveristics). Experimental arenas (single or split in half) were defined to cover the surface of the vials (except the bottom and the stopper). Speed per active fly was calculated every frame (1 sec = 30 frames) and flies were considered active at 5 mm/sec. Data was exported to Excel for statistical analysis.

### Quantitative RT-PCR

To quantify the levels of PrP transcripts expressed from hamster or mouse PrP transgenes, we performed real-time RT-PCR assays using the SYBR green fluorescent reagent. Total RNA was isolated (Trizol, Invitrogen) from fly heads expressing PrP under the control of the OK107 driver. DNA traces were eliminated with Turbo DNAse (Ambion). Real-time PCR reactions were done using the ABI PRISM 7700 system (Applied Biosystems) and the relative amounts of mRNAs were calculated by amplifying RNA Pol II mRNA in the same reactions. For Hsp70 experiments, PrP transcripts were quantified in GFP/PrP or Hsp70/PrP flies. Plotted values were obtained from three independent reactions and arbitrarily normalized against one of the lines tested.

### Tissue Homogenates, Western Blot, and ELISA

Ten to twenty fly heads from each relevant genotype were used for brain extracts. Fly heads were homogenized in 30 µl of PBS containing 150 mM NaCl, 1% Triton X-100, 4 mM EDTA and Complete Protease Inhibitors (Roche). 10% brain homogenates (w/v) from healthy and sick hamsters were prepared as described [46]. Protein extracts were fractionated by SDS-PAGE under reducing conditions, electroblotted into nitrocellulose membranes and probed against 3F4 and β-tubulin (1∶200,000, Sigma) antibodies. To detect fibrillar conformations, the extraction was carried out as above, but without detergent, separated by denaturing PAGE and incubated with the OC antibody at 1∶ 5,000 [20]. For ELISA, 6 µL of fly head homogenate in coating buffer (0.1 M Sodium bicarbonate, pH 9.6) were placed in 96-well plates, 96 µL of coating buffer were added followed by 2 hour incubation at 37°C. After washing and blocking, 100 µL of OC antibody (1∶3000) were added per well and incubated 1 h at 37°C. Wells were washed again followed by an incubation with 100 µL of anti-rabbit HRP (1∶2000) for 1 hour at 37°C. After washing, 100 µL of TMB-1 (KPL) were added to each well and incubated at room temperature. When the color developed, the reaction was stopped with 100 µL of HCl 1 M and read at 450 nm. As positive control we used pre-aggregated amyloid-β fibers using published procedures [20].

### NaPTA/sarkosyl Precipitation

NaPTA precipitation was conducted as outlined [19], except that the final volume was scaled down to 60 µl and equivalent amounts of fly and hamster extracts were processed. Supernatant, pellet and an equivalent aliquot of the total fraction were then analyzed by Western blots.

### Protease-Resistance Assays

Homogenates (Da-Gal/PrP-M6) from young (day 1) and old (day 30) flies were incubated with PK concentrations from 0 to 5 µg/ml for 30 min at 4°C. The digestions were stopped by adding 2 mM PMSF and the samples were resolved in 12% SDS gels, transferred to a .2 µm nitrocellulose membrane and stained for 3F4 immunoreactivity.

### Immunoprecipitation Assays

PrP^Sc^-specific conformations were detected in hamster and fly brain extracts using the 15B3 immunoprecipitation kit (Prionics AG, Switzerland) [22]. For the direct interaction between PrP and Hsp70, Pull-down assays were conducted with 6His-tagged recombinant human Hsp70 from StressMarq (SPR-103B). The ProFound Pull-down PolyHis protein∶protein interaction kit (PIERCE) was used according to the manufacturer, except that bait immobilization to cobalt chelate beads was performed for 4 h and subsequent incubation with PrP-containing extracts was conducted in the presence of 0.25% BSA. Co-immunoprecipitation assays were conducted using Dynabeads M-280 Tosylactivated coupled to the 3F4, Monoclonal Hsp70 (StressGen) or β-Galactosidase (Sigma) antibodies as specified by the manufacturer (Invitrogen). Where indicated, ATP was also added to the binding reactions at 10 mM. Immunoprecipitated proteins were subjected to western blot using anti-human Hsp70 polyclonal antibody (1∶10,000) or 3F4 for the reverse experiments.

### Microsome and Lipid Raft Purification

For microsome preparations, forty heads were homogenized in 70 µL of BIB buffer (320 nm sucrose, 0.5 mM EGTA, 10 mM Tris pH 7.8, 1 mM DTT and 1× protease inhibitor). Samples were centrifuged 30 min at 5,000 rpm at 4°C to eliminate debris. Supernatants were subjected to a second centrifugation for 1 h at 20,000 rpm at 4°C. Supernatants (cytoplasmic fraction) were recovered and pellets (microsomes) were resuspended in BIB buffer to run a Western blot. Detergent-resistant membranes (DRMs) or lipid rafts were prepared as described [48] with few modifications. Briefly, 50 fly heads were lysed in 250 µl of cold TNET buffer (100 mM Tris, pH 7.5, 150 mM NaCl, 2 mM EGTA, 1% Triton X-100, 1× protease inhibitor) using a mini glass homogenizer and incubated in ice for 30 min. After debris removal, 200 µl of crude extract were mixed with 400 µl of 60% Optiprep™ in 5 mL ultracentrifuge tubes and overlaid with 1.8 mL of 30% Optiprep and 600 µl of 5% Optiprep. Gradients were spun in a Sorvall S52-ST rotor at 139,000×*g* for 5 h at 4°C. Ten 300 µl fractions were collected from the top and analyzed by western blotting after methanol precipitation. The anti-α Subunit of the Na+/K+ ATPase (1∶ 100,000) and Syntaxin (1∶ 50) antibodies were obtained from the Developmental Studies Hybridroma Center.

### Statistical Analysis

The significance of the differences between PrP and Hsp70/PrP flies in climbing assays was determined by Chi-square with 7 degrees of freedom (8 points per day). The average speed was tested by a two-tailed t-student. Statistical significance was considered below 1% of chance.

## Supporting Information

Figure S1 Wild type mouse PrP induces locomotor dysfunction. (A) The relative expression of PrP transcripts induced by two wild type mPrP lines was compared with moderate (M9) and strong (M6) HaPrP lines by quantitative RT-PCR. The mPrP-P1 line induces slightly lower expression than HaPrP-M6, but mPrP-J1 induces twice as much PrP transcripts. (B) Wild type mPrP expression induces locomotor dysfunction. Expression of the strong (P1) and very strong (J1) mPrP transgenes in motor neurons (BG380-Gal4) induce early locomotor dysfunction. When compared to HaPrP-M6, the strength of these phenotypes correlate with the expression levels shown in A. Expression of LacZ is used as control.
(0.46 MB TIF)

Figure S2 The Solubility of cytosolic β-Galactosidase is not affected by age. Separation of sarkosyl/NaPTA soluble (S) and insoluble (P) fractions from head extracts of 1 or 40 day-old flies expressing bacterial LacZ. β-Galactosidase is only detected in the soluble fraction in both young and old flies, indicating that its solubility does not change over time.
(0.76 MB TIF)
