# Supplementary figures and images for: In Vivo Generation of Neurotoxic Prion Protein: Role for Hsp70 in Accumulation of Misfolded Isoforms
Source: PLoS Genet. 2009 Jun 5;5(6):e1000507. doi: 10.1371/journal.pgen.1000507 (PMC2683939; doi:10.1371/journal.pgen.1000507)

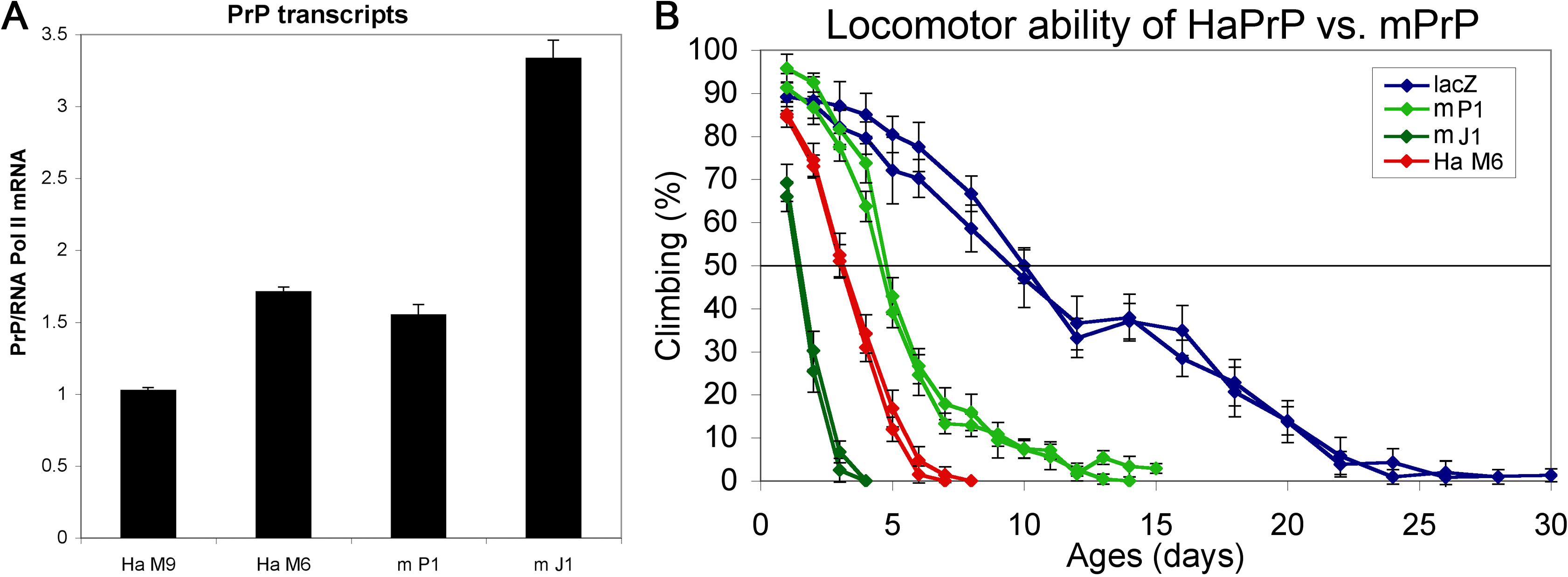

Supplement: Figure S1 — Wild type mouse PrP induces locomotor dysfunction. (A) The relative expression of PrP transcripts induced by two wild type mPrP lines was compared with moderate (M9) and strong (M6) HaPrP lines by quantitative RT-PCR. The mPrP-P1 line induces slightly lower expression than HaPrP-M6, but mPrP-J1 induces twice as much PrP transcripts. (B) Wild type mPrP expression induces locomotor dysfunction. Expression of the strong (P1) and very strong (J1) mPrP transgenes in motor neurons (BG380-Gal4) induce early locomotor dysfunction. When compared to HaPrP-M6, the strength of these phenotypes correlate with the expression levels shown in A. Expression of LacZ is used as control. (0.46 MB TIF) [file pgen.1000507.s001.tif]

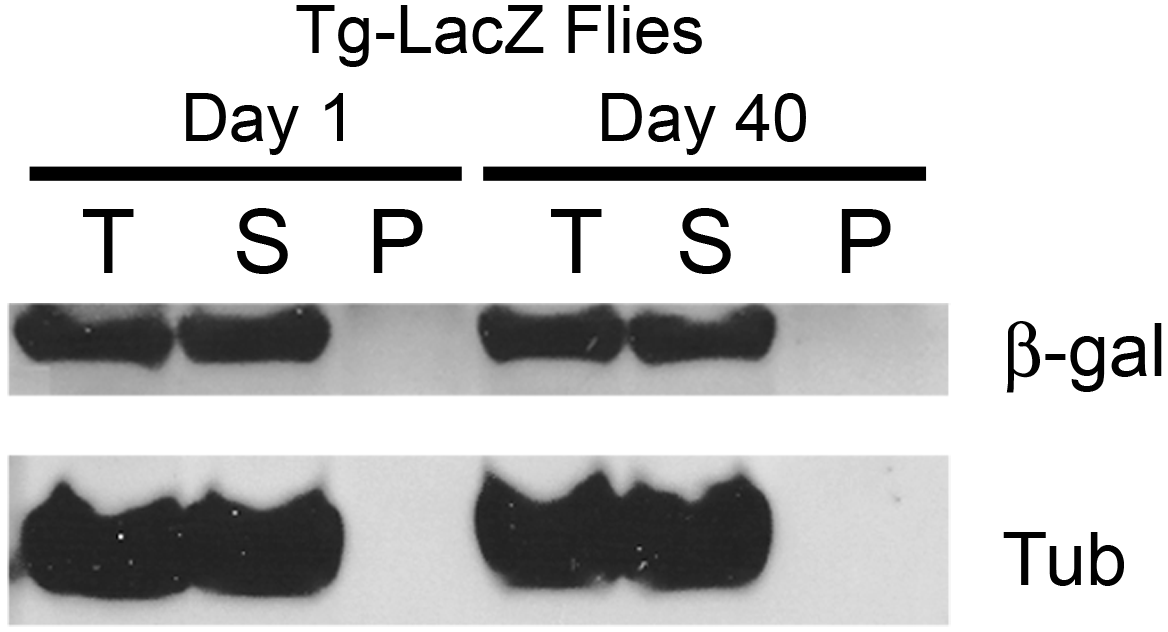

Supplement: Figure S2 — The Solubility of cytosolic β-Galactosidase is not affected by age. Separation of sarkosyl/NaPTA soluble (S) and insoluble (P) fractions from head extracts of 1 or 40 day-old flies expressing bacterial LacZ. β-Galactosidase is only detected in the soluble fraction in both young and old flies, indicating that its solubility does not change over time. (0.76 MB TIF) [file pgen.1000507.s002.tif]
